# Exogenous hydrogen sulfide alleviates surgery-induced neuroinflammatory cognitive impairment in adult mice by inhibiting NO signaling

**DOI:** 10.1186/s12871-019-0927-z

**Published:** 2020-01-09

**Authors:** Lijun Yin, Shunli Gao, Changkun Li

**Affiliations:** Department of Anesthesiology, Baodi Clinical College of Tianjin Medical University, No.8 Guangchuan Road, Baodi District, Tianjin, 301800 China

**Keywords:** POCD, Neuroinflammation, H_2_S, iNOS, NO

## Abstract

**Background:**

To investigate the effect and mechanisms of exogenous hydrogen sulfide in surgery-induced neuroinflammatory cognitive dysfunction.

**Methods:**

C57BL/6 J male mice (*n* = 140) were used and randomly divided into seven groups: the sham group, surgery group, GYY4137 group, L-NAME group, surgery+GYY4137 group, surgery +L-NAME group, and surgery+GYY4137 + L-NAME group. After the interventions, open field tests (OFT) and the Morris water maze (MWM) test were conducted to evaluate learning and memory abilities in the mice. ELISAs, nitrate reductase assays, and Western blots (WB) were conducted to evaluate interleukin-1 beta (IL-1β), tumor necrosis factor-alpha (TNF-α), nitric oxide (NO), inducible nitric oxide synthase (iNOS), malondialdehyde (MDA), and antioxidant enzyme superoxide dismutase (SOD) levels. Furthermore, the expression level of microglial marker ionized calcium binding adaptor molecule 1 (IBA) in the hippocampal CA1 and CA3 areas was detected by an immunohistochemical (IHC) assay and apoptotic cells were observed using terminal deoxynucleotidyl transferase dUTP end-labeling (TUNEL) staining kits.

**Results:**

We found that surgery induced neuroinflammatory cognitive dysfunction, oxidative stress, microglial activation, and cell apoptosis in the hippocampus. Moreover, following surgery, NO and iNOS levels were elevated in the hippocampus. Notably, all the effects caused by surgery were reversed by the H_2_S donor GYY4137 or the iNOS inhibitor N(gamma)-nitro-L-arginine methyl ester (L-NAME). However, the combined application of GYY4137 and L-NAME was not superior to treatment with either agent alone and the effect of GYY4137 was similar to that of L-NAME.

**Conclusion:**

The long-acting hydrogen sulfide donor GYY4137 had an ability to reversed the cognitive deficits and inflammation caused by carotid artery exposure surgery. This implies that NO signaling pathways might participate in this process. These results indicate that exogenous H_2_S may be a promising therapy for POCD.

## Background

Post-operative cognitive dysfunction (POCD) is characterized by cognitive impairments, including learning and memory deficits in patients after anesthesia and surgery, and affects about 30% of young and elderly patients after hospital discharge [1–3]. In addition to negative factors such as impaired communication and reduced daily activities, life quality, and work performance, POCD is related to higher morbidity and mortality, longer hospitalization, and greater economic spending [3–6]. Aging, education level, surgical trauma, anesthesia, post-operative analgesia, and infection are risk factors for developing POCD [7–9]. Notably, structural and biochemical alterations, such as reduced neurogenesis and the induction of neuroinflammation in the hippocampus, an area of the brain mainly responsible for cognition that is highly vulnerable to aging, are most likely the mechanisms underlying POCD [10–13]. Animal studies suggest that neuroinflammation may be a major cause of anesthesia and surgery-induced cognitive impairment [14, 15]. Nevertheless, the causes and pathogenesis of POCD are yet to be fully investigated.

Nitric oxide (NO) plays a crucial role in supporting normal physiological functions, but pathological conditions such as inflammation can stimulate the production of high concentrations of NO, which may initiate neurodegeneration [16]. Moreover, NO is involved in cellular modifications such as microglial activation, neuronal cell apoptosis, and oxidative stress, and overproduction of NO impairs cognitive function. The bioavailability of NO is recognized as a predictive risk factor for Alzheimer’s disease (AD) and early POCD [17–19]. NO is synthetized by nitric oxide synthases (NOS), which are grouped into three classes: endothelial NOS (eNOS), neuronal NOS (nNOS), and inducible NOS (iNOS). eNOS and nNOS are associated with endothelial and neuronal cells, respectively, and produce low and transient concentrations of NO. The production of iNOS is not restricted to specific cells but is expressed following different stimuli such as inflammation, and is a major pro-inflammatory and destructive mediator in inflammatory disease [20]. iNOS also promotes synaptic plasticity and brain function deficits, such as cognitive deficits [17, 21]. L-nitro-arginine methyl ester (L-NAME), an inhibitor of NOS that can inhibit NO biosynthesis, can attenuate brain dysfunction [22]. Therefore, NO may represent a pathogenic signal for POCD.

Hydrogen sulfide (H_2_S), the third gaseous transmitter alongside NO, can regulate the release of NO and its interaction with NO in the hippocampus can alleviate brain dysfunction [22–24]. In addition, H_2_S exerts both pro- and anti-inflammatory effects [24]. The exogenous hydrogen sulfide donor GYY4137 induces the slow release of H_2_S and reduces intestinal inflammation and protects against intestinal ischemia via eNOS-dependent pathways [25].

In the present work, we investigated the effect and mechanism of action of the H_2_S donor GYY4137 in a surgery-induced neuroinflammatory cognitive deficit model in adult mice.

## Methods

### Animals and groups

We used 140 healthy 8-week-old adult male C57BL/6 J mice weighing 25–30 g, purchased from the Animal Experiment Center of the Institute of Radiation Medicine of the Chinese Academy of Medical Science. All mice were maintained for at least 1 week in a temperature-controlled room at 25 °C and 60% relative humidity with a 12 h light cycle and given free access to a standard laboratory diet and water before experiments. The mice were treated according to the ethical guidelines of the Animal Experiment Center of the Institute of Radiation Medicine of the Chinese Academy of Medical Science, and the Animal Studies Committee of the Animal Experiment Center of the Institute of Radiation Medicine of the Chinese Academy of Medical Science approved the experimental protocol. Mice were randomly divided into seven groups: sham group (all surgical procedures were performed without right carotid artery exposure on Day 0), surgery group (right carotid artery exposure on Day 0), GYY4137 group (intraperitoneal injection of 50 mg/kg GYY4137 in phosphate-buffered saline (PBS) 1 h before sham surgery), L-NAME group (water intake with 15 mg/kg L-NAME in the drinking water from Day 1 before sham surgery to Day 3 post-surgery), surgery+GYY4137 group (intraperitoneal injection of 50 mg/kg GYY4137 1 h before carotid artery exposure), surgery+L-NAME group (water intake with 15 mg/kg of L-NAME before carotid artery exposure up to Day 3 post-surgery), and surgery+GYY4137 + L-NAME group. Each group included 20 mice.

### Right carotid artery exposure-induced surgical inflammatory injury model

After mice were anesthetized with 3% sevoflurane, a right carotid artery exposure surgery procedure was carried out, as previously described [1, 26]. After about 10 min of the total surgical procedure was performed, all animals received compound lidocaine cream containing 25 mg prilocaine and 25 mg lidocaine (Beijing Ziguang Medication Manufacture Corporation Ltd., China) on the wound.

### Open field test (OFT)

On the second day post-surgery, the OFT was performed to determine the spontaneous locomotor activity of mice, as previously described [26]. Briefly, a plastic open field box (50 × 50 × 23 cm) with 16 equal sector bases was used. Mice were placed at the corner for 5 min of acclimatization, and in the next 5 min, while all four paws of the animals were placed in a new square, the number of crossings were recorded and raising of the forepaws was recorded as the number of rears. During the test procedure, 5% ethyl alcohol was employed for cleaning the box to remove animal cues.

### Morris water maze (MWM)

On Days 3–8 post-surgery, the MWM test was conducted to evaluate learning and memory ability. The experiment was performed using water maze equipment (Jiliang software (DigBehv) technology company, Shanghai, China) from 9:00–12:00 am every day over a total of 6 days, as previously described [26, 27]. The first 5 days were for directional navigation to test the learning ability of mice. Two indicators were recorded: one was the time it took the mouse to find the platform, named “Escape Latency,” and the second was the distance it took to find the platform, named “Swimming Distance (Path Length).” When the MWM was performed, the mouse was placed into the pool facing the wall. Then, the time and distance were recorded from when/where the mouse was placed into the water until it found the platform. If more than 60 s passed without it finding the platform, the mouse was guided to arrive to the platform and allowed to rest for 10 s on the platform and the escape latency was recorded as 60 s. The memory ability of mice was tested on the last day using space exploration. The platform was removed, and the mouse was placed in the water twice facing to the pool wall. Then, the time and distance of swimming in the third quadrant (target quadrant) was recorded.

### Hippocampal tissue preparation

Mice from each group were deeply anesthetized with 3% sevoflurane at 1 (*n* = 8), 4 (*n* = 6) and 8 (*n* = 6) days post-surgery. Mice were decapitated and hippocampi were isolated from the brain on ice and stored at − 80 °C prior to use in the following experiments.

### Enzyme-linked immunosorbent assay (ELISA)

The concentrations of interleukin-1β (IL-1β) and tumor necrosis factor α (TNF-α) were measured in hippocampal tissues on Days 1, 4, and 8 post-surgery using ELISA, following the manufacturer’s instructions. The specific steps employed and description are consistent with a previous study [26]. For quantification, the concentrations of IL-1β and TNF-α were calculated based on the 450 nm wavelength absorbance on a spectrophotometer and expressed as pg/mg protein.

### NO concentration detection

On Day 1 post-surgery, the NO concentration in hippocampal tissues was detected using the nitrate reductase method, according to a previous report [28]. Briefly, the operation sequence complied with the manufacturer’s instructions for the commercially available NO Fluorometric Assay Kit (Nanjing jiancheng biology co. LTD, Nanjing, China). The concentration of NO is presented as nmol/mg protein and was calculated using the 550 nm absorbance wavelength.

### Western blot (WB)

WB was performed to evaluate iNOS protein content in hippocampal tissues at 24 h post-surgery. Briefly, protein was extracted from the hippocampus then treated with RIPA lysis buffer (Beyotime, China) for 30 min and centrifuged at 14,000×*g* for 30 min. Equal amounts of protein were separated by 10% sodium dodecyl sulfate-polyacrylamide gel electrophoresis and transferred to a polyvinylidene difluoride membrane (Millipore). After blocking with 5% milk in Tris buffered saline containing 0.05% Tween-20 (TBS-T) at room temperature for 1 h, the membrane was incubated at 4 °C overnight with the primary iNOS antibody (1:500, Ab15323, Abcam, Cambridge, MA), and an anti-β-tubulin antibody (1:500, Ab6046, Abcam, Cambridge, MA) was used as a control. After washing with TBS-T and further incubation with the secondary antibody, horse radish peroxidase (HRP)-conjugated anti-rabbit IgG (1:1000, A0545, Sigma-Aldrich, St. Louis, MO), at 37 °C for 2 h, the blots were visualized using an enhanced chemiluminescence (ECL) reagent.

### Oxidative stress detection

Twenty-four hours post-surgery, the concentrations of the oxidative product malondialdehyde (MDA) and antioxidant enzyme superoxide dismutase (SOD) were measured in hippocampal tissues using appropriate kits from Nanjing Jiancheng Bioengineering Institute (A006–2, A001–3), according to the manufacturer’s instructions.

### Immunohistochemistry (IHC)

The microglial marker ionized calcium binding adaptor molecule 1 (IBA-1) was detected in the hippocampal cornu ammonis (CA) 1 and CA3 1 day post-surgery using IHC staining, as previously described [29, 30]. Briefly, hippocampal tissues were harvested and post-fixed. Sections of 4-μm thickness were cut using a freezing microtome (Leica CM1900, Germany), collected in a 24-well plate, and rinsed. After immersing in 0.01 M PBS containing 5% goat non-immune serum and 0.3% TritonX-100 solution at 37 °C for 30 min, the slices were subsequently incubated for 48 h at 4 °C with 2% goat serum containing the goat polyclonal primary antibody anti-IBA-1 (1:100; WAKO). The slices were washed and incubated in Reagents I and II from the Reagent Kit (Chemicon, Anti-Rabbit/Mouse Poly-HRP IHC Detection Kit, USA) for 30 min each at 37 °C. The slices were again rinsed five times, each for 5 min in 0.01 M PBS-T. Finally, sections were detected using 3,3′-Diaminobenzidine (DAB) staining. A negative control was generated by replacing the primary antibody with 2% goat serum to ascertain the specificity of antibody staining. Immunoreactive products were observed and photographed with a light microscope (Leica. DMIRB, Germany) coupled with a computer assisted video camera.

### Tunel staining

Tunel staining was performed on Day 1 post-surgery to detect apoptotic cells in the hippocampal CA1 and CA3. The procedure was as follows: after freezing, 50 μl of freshly diluted proteinase K was added to the tissue at a concentration of 20 μg/ml (147 μl of 10 mM Tris-HCl added to 3 μl 1 mg/ml proteinase K). Digestion was performed for 15 min at 37 °C, followed by washing in 0.01 M PBS three times for 5 min each. Then, the slices were rinsed using 0.1% diethyl pyrocarbonate (DEPC) water at room temperature for 30 min. PBS (0.01 M) was used to wash the sections three times for 5 min each. Redundant fluid was removed from the slices. Solution I and Solution II were added (1:9 volume ratio, prepared on PE gloves on ice, well distributed). The Tunel reaction mixture was prepared. Subsequently, freshly prepared 3% H_2_O_2_-methanol was added and incubated with the sections at room temperature for 15 min, followed by washing with 0.01 M PBS three times for 5 min each. Confining liquid (50 μl 5% bovine serum albumin) was added and sections were incubated for 30 min at 37 °C. Then, the confining liquid was removed without washing. Transforming agent-AP (50 μl) was added to each slice. Then, the slices were placed in the wet box and incubated for 40 min at 37 °C followed by washing with 0.01 M PBS three times for 5 min each. TVBT was used for color production at room temperature. A general light microscope was used to observe the color for 5–10 min. Distilled water was used to stop the color reaction. The sections were washed under flowing water for 10 min to clean the NBT grains. Conventional dehydration and mounting were performed. A light microscope was used to observe the result of Tunel staining. In the negative control group, Solution II was added without Solution l. The sample slices were covered with a plastic cap. Then, the samples were placed into a wet box, labeled for 1 h at 37 °C and then overnight at 4 °C for more than 20 h. Then, samples were washed with 0.01 M PBS three times for 3 min each. In the positive control group, Dnase l was added and incubated at room temperature for 10 min. Lastly, the numbers of cells undergoing apoptosis and the morphological features were observed.

### Statistical analysis

Data were analyzed using SPSS 17.0 software and expressed as the mean ± standard deviation (SD). Comparisons between multiple groups were performed with one-way analysis of variance (ANOVA) or a two-way ANOVA with a Bonferroni’s multiple comparisons post-hoc test where appropriate. Data from the MWM test were analyzed using a two-way ANOVA (treatment × trial time) with repeated measures (trial days). Differences were considered to be statistically significant at *P* < 0.05.

## Results

### Exogenous H_2_S reverses impaired cognitive function without altering the locomotor dysfunction caused by surgical injury, and is similar to L-NAME

**Fig. 1:**
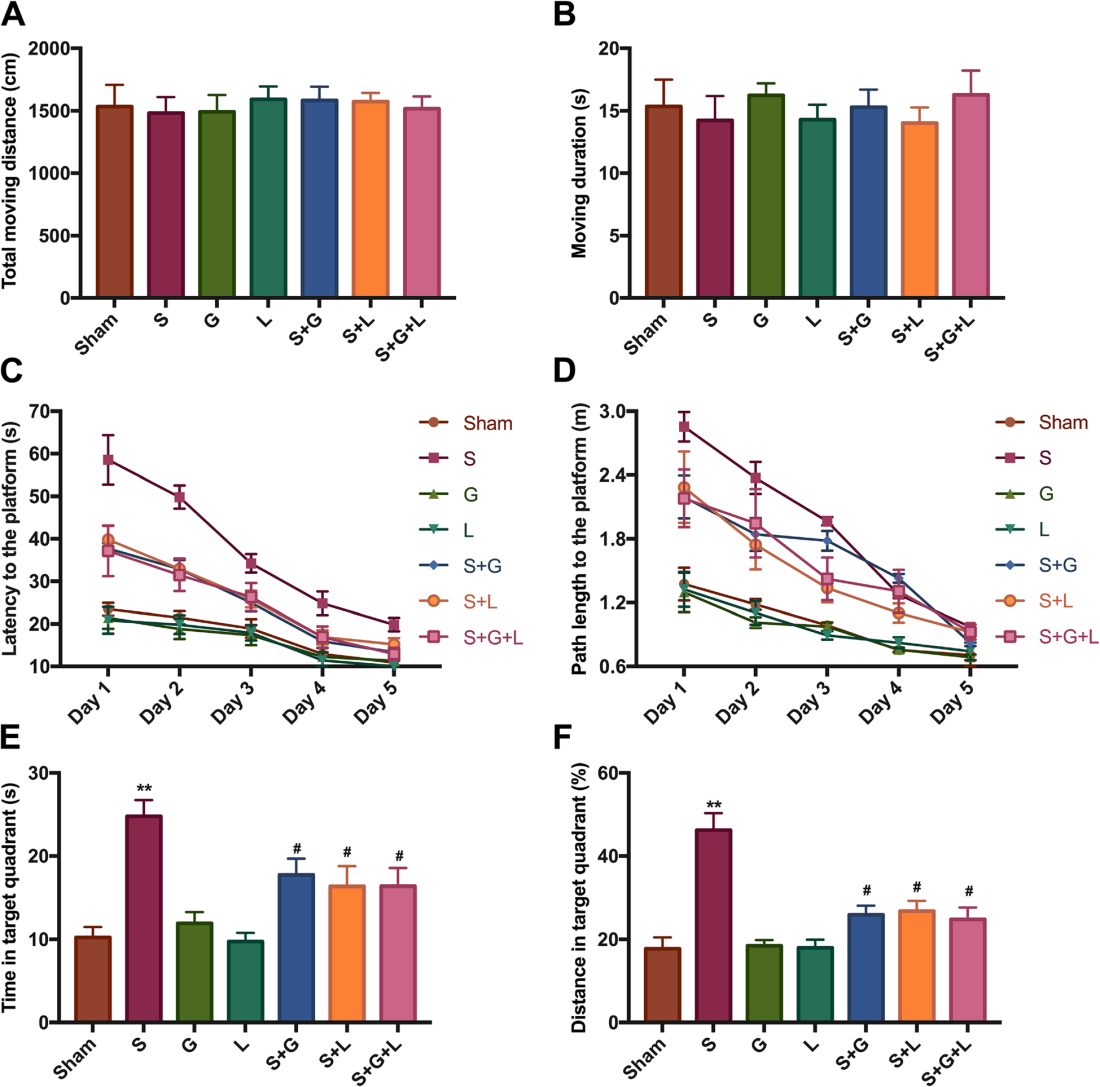
Exogenous H_2_S reverses impaired cognitive function without altering locomotor performance, which is similar to L-NAME. **a** and **b** Locomotor activity was assessed using the OFT on Day 2 post-surgery and the total moving distance and moving duration were measured. **c** and **d** Spatial learning ability was evaluated using the MWM on Days 3 to 7 post-surgery, and the escape latency and path length were measured. **e** and **f** Spatial memory capacity was assessed using the MWM on Day 8 post-surgery, and the percent time and length in the target quadrant were measured. * *P* < 0.05, compared with the sham group; ** *P* < 0.01, compared with the sham group; # *P* < 0.05, compared with the surgery group; ## *P* < 0.01, compared with the surgery group

In our study, cognitive performance was assessed using the OFT and MWM. In the OFT test, the total moving distance and moving duration data showed no visible difference in locomotor activity between the groups (Fig. 1a, b). MWM outcomes showed that surgery reduced spatial learning, with a longer escape latency and path length and decreased memory ability with a reduced percent time and length in the target quadrant when compared to the sham group. Compared with the sham group, the exogenous H_2_S donor GYY4137 significantly improved spatial learning and memory capacity, and, compared with the surgery group, GYY4137 could partially reverse the effects of surgical injury. The NOS inhibitor L-NAME alone did not influence learning and memory performance compared with the sham group, but compared with the surgery group it also partially reversed surgery-induced learning and memory decline. The effect was similar to GYY4137. Meanwhile, the combination of GYY4137 and L-NAME did not exert additional advantageous effects compared with GYY4137 or L-NAME alone (Fig. 1c–f). These findings suggest that the cognitive deficits in spatial learning and memory were not owing to a reduction in spontaneous locomotor activity.

### Exogenous H_2_S reverses surgery-induced neuroinflammation on day 1 and day 4 post-surgery, but not on day 8 post-surgery, which may be related to the reduction in NO and iNOS concentrations in the hippocampus, and the effect is similar to L-NAME

**Fig. 2:**
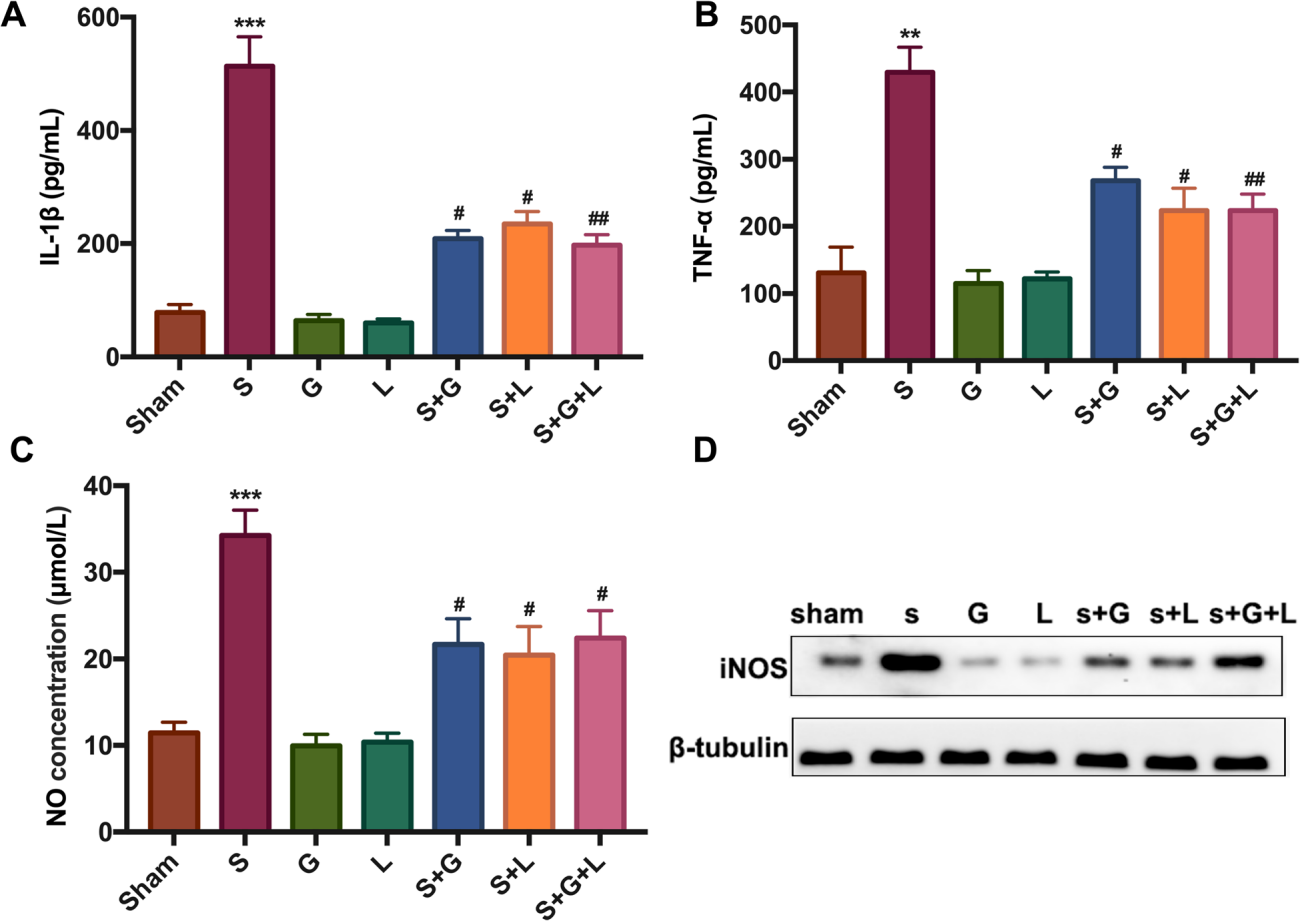
Exogenous H_2_S reverses surgery-induced neuroinflammation on Days 1 and 4 post-surgery but not on Day 8 post-surgery, which may be related to reduced hippocampal NO and iNOS. **a** and **b** Concentrations of IL-1β and TNF-α in the hippocampus were detected by ELISA on Days 1, 4, and 8 post-surgery. **c** Concentrations of hippocampal NO were measured using the nitrate reductase method at 24 h post-surgery. **d** Hippocampal iNOS was detected by WB at 24 h post-surgery. IL-1β, interleukin-1β; TNF-α, tumor necrosis factor α; NO, nitric oxide; iNOS, inducible nitric oxide synthase. * *P* < 0.05, compared with the sham group; ** *P* < 0.01, compared with the sham group; # *P* < 0.05, compared with the surgery group; ## *P* < 0.01, compared with the surgery group

Surgery induced marked neuroinflammation in the hippocampus with increased concentrations of IL-1β and TNF-α noted, compared to the sham group. The highest concentration appeared on Day 1 post-surgery, and the neuroinflammation was alleviated by Day 4 post-surgery. The neuroinflammation status was substantially restored on Day 8 post-surgery. No significant difference was noted between groups. Both GYY4137 and L-NAME could inhibit the surgery-induced neuroinflammation, and the combination of GYY4137 and L-NAME did not produce a better effect compared with GYY4137 or L-NAME alone (Fig. 2a, b). Furthermore, we detected the NO and iNOS in the hippocampus at 24 h post-surgery, and the results were consistent with those for IL-1β and TNF-α (Fig. 2c, d).

### Exogenous H_2_S reverses surgery-induced oxidative stress, microglial activation, and cell apoptosis in the hippocampus, which is similar to L-NAME

**Fig. 3:**
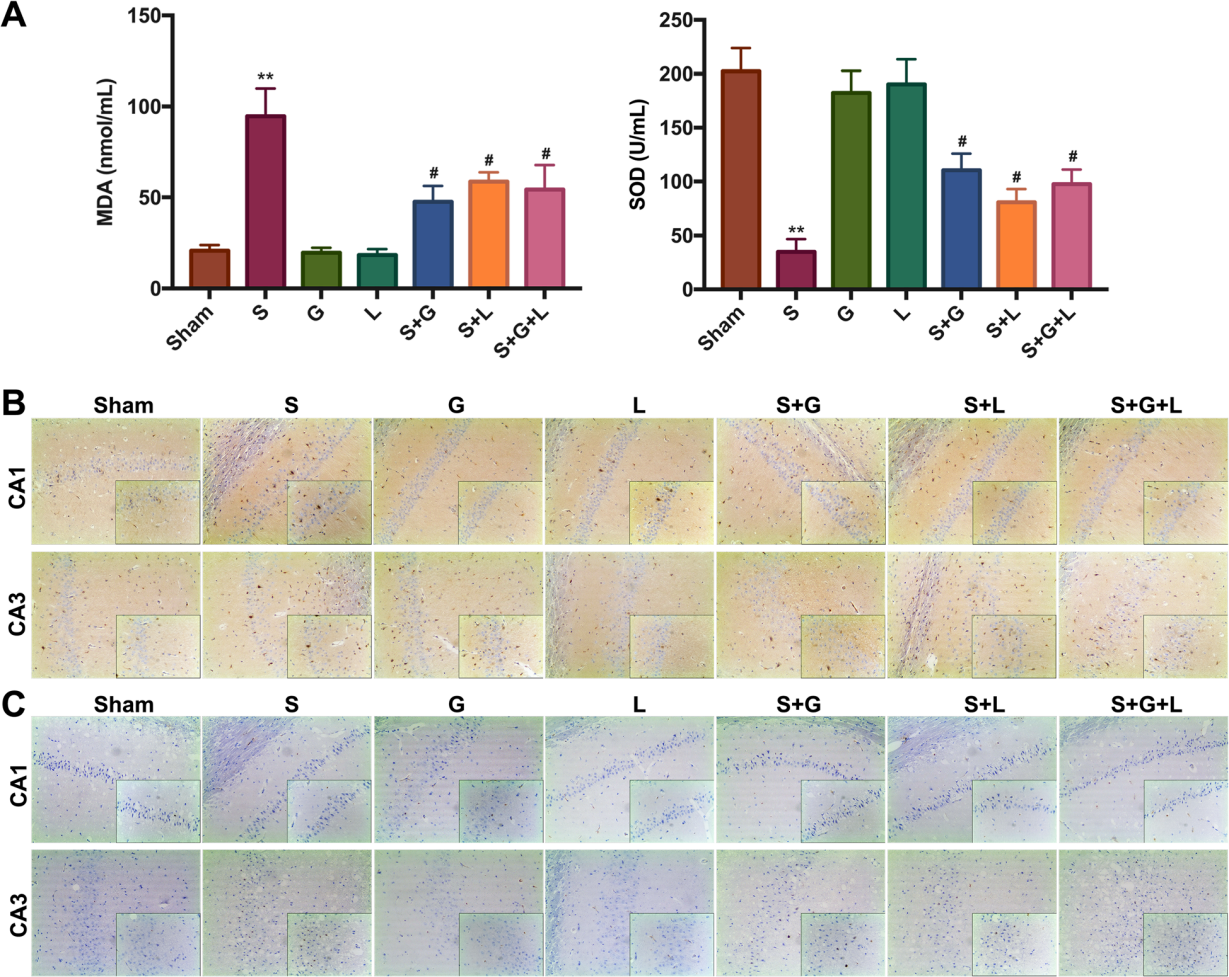
Exogenous H_2_S reverses surgery-induced oxidative stress, microglial activation, and cell apoptosis in the hippocampus, which is similar to L-NAME. **a** The oxidative product MDA and antioxidant enzyme SOD were measured in hippocampal tissue. **b** The microglial marker IBA-1 (brown) was detected in the hippocampal CA1 and CA3 regions on Day 1 post-surgery using IHC staining. Scale bars: × 100, 200 μm; × 200, 100 μm. **c** Neuronal apoptosis in the hippocampal CA1 and CA3 regions was detected on Day 1 post-surgery using TUNEL staining. Scale bars: × 100, 200 μm; × 200, 100 μm. * *P* < 0.05, compared with the sham group; # *P* < 0.05, compared with the surgery group

Twenty-four hours post-surgery, oxidative stress, microglial activation, and cell apoptosis were assessed using MDA and SOD kits, IHC, and Tunel staining, respectively. The results show that surgery induced an oxidative stress reaction in the hippocampus, with increased production of MDA and decreased production of SOD noted. Treatment with GYY4137 or L-NAME mitigated the degree of oxidative stress. However, GYY4137 or L-NAME alone had no effect. Moreover, the combination of GYY4137 and L-NAME had no additional advantage, compared with GYY4137 or L-NAME alone (Fig. 3a). Additionally, more microglial and apoptotic cells were found in the surgery group compared to the sham group. Mice treated with GYY4137 or L-NAME showed attenuation of microglial activation and cell apoptosis in the hippocampal CA1 and CA3. There were no marked differences between the sham group and GYY4137 group, or between the sham group and L-NAME group. Moreover, the combination of GYY4137 and L-NAME was not significantly better than GYY4137 or L-NAME alone (Fig. 3b, c). These data indicate that the protective effects of GYY4137 are similar to L-NAME.

## Discussion

In this study, we identified that the H_2_S donor GYY4137 plays a protective role in surgery-induced neuroinflammatory cognitive disorder in adult mice by suppressing NO and iNOS in the hippocampus. Right carotid artery exposure surgery caused an increase in the pro-inflammatory cytokines IL-1β and TNF-α in the hippocampus, and impaired spatial learning and memory ability, but not locomotor activity. The increase in hippocampal NO and iNOS, accumulation of the oxidative product MDA, and reduction in the antioxidant enzyme SOD in the hippocampus, microglial activation, and cell apoptosis in the hippocampal CA1 and CA3 were all remedied by the H_2_S donor GYY4137 or iNOS inhibitor L-NAME. Moreover, the combined application of GYY4137 and L-NAME was not superior to their individual application, and the effect of GYY4137 was similar to L-NAME.

Clinical data have shown that anesthesia and surgical trauma are the main causes of POCD, which may increase morbidity and mortality [3]. In addition, surgery-induced neuroinflammation in the hippocampus is closely related to POCD [26]. Diverse surgical operations performed on animals have been adopted to mimic POCD [8, 31, 32]. Wang et al. demonstrated that right carotid artery exposure induced apparent cognitive impairment in spatial learning and memory, as detected using the MWM test, and reduced freezing time in the FCS experiment, but did not impair locomotor activity in the OFT or attenuate the increase in the pro-inflammatory cytokines IL-1β, IL-6, and TNF-α in the hippocampus on Day 4 post-surgery in aged C57BL/6 J male mice [26]. Another previous study also reported that carotid artery exposure induced POCD and a neuroinflammatory response, as well as reduced neurogenesis in adult C57BL/6 J mice. Our study used adult C57BL/6 J mice and mimicked the surgery model used by Wang et al. to obtain consistent results. Notably, the H_2_S donor GYY4137 and iNOS inhibitor L-NAME effectively ameliorated the surgery-induced neuroinflammation and cognitive impairment.

NO is generated excessively following inflammatory stimulation and may induce neurodegeneration and accelerate cognitive decline and dementia [33]. NO is produced by NOS, and iNOS expression is not restricted to specific cells and represents a major pro-inflammatory and destructive mediator in inflammatory disease [20]. The suppression of iNOS plays a neuroprotective role, including reducing traumatic brain injury-induced synaptic plasticity and cognitive function deficits in male C57BL/6 J mice [21]. In our work, surgery induced distinct neuroinflammation in the hippocampus and stimulated a high level of iNOS expression, which promoted NO oversynthesis in the hippocampus. However, treatment with GYY4137 or L-NAME reversed the inflammation-induced overproduction of iNOS and NO in the hippocampus. The combined application of GYY4137 and L-NAME did not produce additional beneficial effects, and the effect of GYY4137 was similar to L-NAME. These results indicate that exogenous H_2_S alleviates surgery-induced neuroinflammatory cognitive impairment in adult mice through the inhibition of NO signaling in the hippocampus. Endogenous and exogenous H_2_S has been shown to exert anti-inflammatory effects that induce recovery from inflammatory disease, such as colitis [34]. A previous study suggested that exogenous H_2_S relieves spatial memory impairments in AD [35]. Exogenous H_2_S also been demonstrated to have neuroprotective effects against brain edema, neurobehavioral function, including learning and memory, and neuronal cell death following subarachnoid hemorrhage [36]. Furthermore, H_2_S can regulate the release of NO, and its interaction with NO in the hippocampus can alleviate brain dysfunction [22–24]. Here, we are the first to demonstrate that exogenous H_2_S ameliorates spatial learning and memory deficits in a surgery-induced POCD mouse model by inhibiting NO and reducing iNOS levels in the hippocampus.

In the current study, we also showed that exogenous H_2_S reverses surgery-induced oxidative stress, microglial activation, and cell apoptosis in the hippocampus, which is similar to the effects of L-NAME. A previous study suggested that oxidative stress is highly connected with reduced cognition, including declined psychomotor speed, mental flexibility, and attention in patients with type 2 diabetes [37]. Oxidative damage in the hippocampus is also implicated in chemotherapy-induced cognitive impairment [38]. Besides, oxidative stress could mediate inflammation and apoptosis in hippocampus, which promote hippocampal lesions [39]. Importantly, microglial activation is a hallmark of the onset of neuroinflammation, and contributes to collateral brain injury [40]. Microglial activation plays a pivotal pathogenic role in the initiation of cognitive disorder-related illness, such as AD [41]. Interestingly, the present evidence indicates that autophagy, apoptosis, inflammation, and oxidative stress play a possible role in the pathogenesis of neurodegenerative disorders that are characterized by a loss of neuronal function [42]. Additionally, NO induces neuronal modifications, for example, microglial activation, neuronal cell apoptosis, and oxidative stress, and high concentrations of NO are harmful to cognitive performance. The bioavailability of NO is recognized as a predictive risk marker for AD and early POCD [17–19]. Therefore, the surgery-induced inflammatory response, which induces high levels of iNOS expression in the hippocampus, then causes NO overproduction in the hippocampus followed by the promotion of oxidative stress, microglial activation, neuronal cell apoptosis, and hippocampal lesions eventually induce the development of POCD. Treatment with GYY4137 or L-NAME can partially restore impaired cognitive function.

## Conclusion

In summary, we found that the long-acting hydrogen sulfide donor GYY4137 had an ability to reversed the cognitive deficits and inflammation caused by carotid artery exposure surgery. This implies that NO signaling pathways might participate in this process. The results indicate that exogenous H_2_S may be a promising therapy for POCD.

## Data Availability

The datasets used and/or analysed during the current study are available from the corresponding author on reasonable request.

## Abbreviations

AD: Alzheimer’s disease
ANOVA: One-way analysis of variance
CA: Cornu ammonis
DAB: Diaminobenzidine
DEPC: Diethyl pyrocarbonate
ECL: Enhanced chemiluminescence
ELISA: Enzyme-linked immunosorbent assay
eNOS: Endothelial NOS
H_2_S: Hydrogen sulfide
HRP: Horse radish peroxidase
IBA-1: Ionized calcium binding adaptor molecule 1
IHC: Immunohistochemistry
IL-1β: Interleukin-1β
iNOS: Inducible NOS
L-NAME: L-nitro-arginine methyl ester
MDA: Malondialdehyde
MWM: Morris water maze
nNOS: Neuronal NOS
NO: Nitric oxide
NOS: Nitric oxide synthases
PBS: Phosphate buffered saline
POCD: Post-operative cognitive dysfunction
SD: Standard deviation
SOD: Superoxide dismutase
TBS-T: Tris buffered saline containing 0.05% Tween-20
TNF-α: Tumor necrosis factor α
WB: Western blot
